# Computational analysis of value learning and value-driven detection of neutral faces by young and older adults

**DOI:** 10.3389/fpsyg.2024.1281857

**Published:** 2024-05-23

**Authors:** Shushi Namba, Akie Saito, Wataru Sato

**Affiliations:** ^1^Psychological Process Team, Guardian Robot Project, RIKEN, Kyoto, Japan; ^2^Department of Psychology, Hiroshima University, Hiroshima, Japan

**Keywords:** facial expression detection, associative learning, diffusion model, emotional value, gerontology

## Abstract

The rapid detection of neutral faces with emotional value plays an important role in social relationships for both young and older adults. Recent psychological studies have indicated that young adults show efficient value learning for neutral faces and the detection of “value-associated faces,” while older adults show slightly different patterns of value learning and value-based detection of neutral faces. However, the mechanisms underlying these processes remain unknown. To investigate this, we applied hierarchical reinforcement learning and diffusion models to a value learning task and value-driven detection task that involved neutral faces; the tasks were completed by young and older adults. The results for the learning task suggested that the sensitivity of learning feedback might decrease with age. In the detection task, the younger adults accumulated information more efficiently than the older adults, and the perceptual time leading to motion onset was shorter in the younger adults. In younger adults only, the reward sensitivity during associative learning might enhance the accumulation of information during a visual search for neutral faces in a rewarded task. These results provide insight into the processing linked to efficient detection of faces associated with emotional values, and the age-related changes therein.

## 1 Introduction

The ability to efficiently determine the emotional significance of facial expressions is crucial for adaptive behavior in social interactions (Stins et al., 2011; Heerdink et al., 2015). Efficient detection of faces with positive emotional value is important for social behaviors and relationships. Similarly, rapid detection of faces displaying negative, threatening emotions can enable an individual to avoid a dangerous situation and preserve resources.

The adaptive qualities of emotional expressions appear to influence attention (Hansen and Hansen, 1988; Fenske and Raymond, 2006; Craig et al., 2014). However, this idea has led to several controversies in the field of psychology (Puls and Rothermund, 2018; Tannert and Rothermund, 2020). For example, there is conjecture regarding the physical and emotional significance of faces providing emotional information (Horstmann et al., 2006; Calvo and Nummenmaa, 2008; Horstmann et al., 2012). To control for perceptual properties when investigating the detection of faces with emotional information, Saito et al. (2022a) employed inherently neutral faces associated with positive or negative values as target stimuli in a visual search task. They based their paradigm on an associative learning task in which neutral stimuli (e.g., colors) were linked with a reward or punishment (Anderson et al., 2011; Wentura et al., 2014; Muller et al., 2016). They found that the reaction time (RT) during a visual search for neutral faces associated with a reward or punishment was reduced compared with that for neutral faces not associated with feedback. In other words, emotional significance facilitated attentional capture during a visual search task.

Given the aging of many contemporary societies, the cognition of older adults has been a major research focus (Ziaei and Fischer, 2016). Older people exhibit a “positivity effect,” i.e., they tend to focus their attention on pleasant stimuli (Reed et al., 2014). However, studies on this topic have yielded inconsistent results. For example, a recent study (Saito et al., 2020) found that both young and older participants readily attended to angry facial expressions. In contrast, older participants did not show this tendency for happy facial expressions. In addition, in an associative learning task combined with a visual search paradigm, Saito et al. (2022b) found that positive (reward) and negative (punishment) outcomes in the associative learning task facilitated attention in the visual search task for successful young and older learners, although there were no differences in emotional valence.

Although previous studies have demonstrated rapid detection of neutral faces associated with an emotional value (Saito et al., 2022a,b), the mechanism underlying the relationship between associative learning and visual search attention remains unclear. Visual search performance has been investigated in successful and unsuccessful learners. However, several scholars have pointed out that such binary groupings are statistically undesirable (DeCoster et al., 2009; Reinhart, 2015). The RT, as a dependent variable, is inherently right-skewed. Analysis of variance (ANOVA), which assumes a normal data distribution, is also inappropriate for this type of study. Another problem is that classification errors are generally analyzed separately from RT, making it impossible to assess the speed-accuracy trade-off. Furthermore, the RTs regarding the relationship between associative learning rate and visual search performance were not compared between the groups because the groups were distinguished using a binary classification scheme based on the associative learning performance (Saito et al., 2022a,b).

Computational modeling using behavioral data has potential for elucidating the mechanisms of human psychological processes (Lee and Wagenmakers, 2014). The reinforcement learning model (Rescorla and Wagner, 1972; Hertwig et al., 2004) can be used to quantitatively evaluate learning rate parameters in learning tasks, and to reveal their associations with other variables and obtain insight into the mechanisms of human behaviors (e.g., Dombrovski et al., 2010; Kwak et al., 2014; Suzuki et al., 2023). Given the above findings, reinforcement learning is likely to be useful for examining performance in learning tasks that involve associative learning; they allow the identification of factors that contribute to success in learning tasks, such as sensitivity to feedback ( η ) or reliance on a model ( τ ).

The diffusion model (Ratcliff, 1978; Ratcliff and Rouder, 1998; Ratcliff and McKoon, 2008; Forstmann et al., 2016) can be used to describe the distribution of RTs associated with the detection of emotional faces (Tipples, 2019; Sawada et al., 2022). The diffusion model includes four main parameters. The threshold separation ( α ) is the distance between two choices (i.e., target presence and absence), z denotes the starting point (which is related to prior bias in two-choice tasks), and ν is the drift rate (speed with which evidence is accumulated in relation to a specific response, i.e., toward the upper or lower threshold). The non-decision time (t0) is based on all time components unrelated to the information accumulation process. RTs tend to be classified as fast or slow in experimental tasks. However, more in-depth metrics can be obtained, such as the speed with which information is accumulated ( ν ), the amount of information required ( α ), and the time required to arrive at a judgement (t0). Analysis of such metrics can reveal the mechanisms underlying psychological processes. Indeed, Sawada et al. (2022) used the diffusion model to estimate cognitive parameters in a visual search task requiring participants to detect angry and happy expressions, and their anti-expressions, within a crowd of neutral faces. Regardless of valence, ν for emotional facial expressions was rapid, α values were large, and t0 values were small. These results suggest that efficient detection of facial expressions is characterized by the faster and more cautious accumulation of information through enhanced attentional allocation.

When investigating the relationships among different behavioral task performance indices, a computational modeling approach can be used to maximize the amount of information obtained. By fitting a reinforcement learning model to associative learning data and a diffusion model to visual search data, and then examining the relationship between the resulting parameters, further insight can be obtained into the mechanisms underlying the detection of faces with emotional meaning.

**Figure 1:**
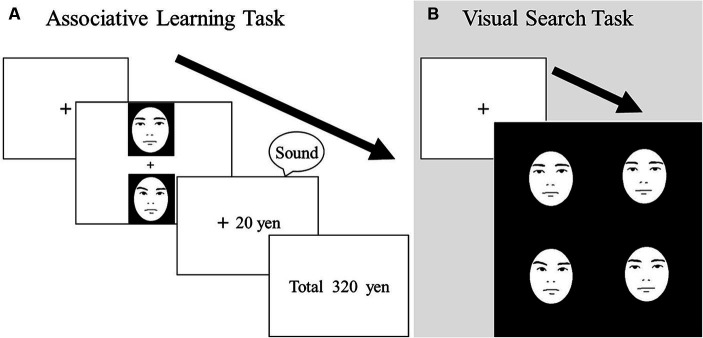
Schematic illustrations of trials in the learning task **(A)** and visual search task **(B)**. In the learning task, participants chose one face from each pair to maximize their earnings. In the visual search task, participants identified one discrepant face embedded among distractor faces. Actual stimuli were photographic faces.

In this study, we explored the psychological processes underlying the rapid detection of faces with emotional meaning by investigating the relationship between associative learning and visual search data. Moreover, we investigated developmental changes by comparing young and older participants. We applied the hierarchical reinforcement learning and diffusion models to data collected in previous studies (Saito et al., 2022a,b) (Figure 1). Then, we checked the relationships between the resulting parameters using both models. Learning success in associative learning tasks was quantitatively represented by changes in learning rates on a continuum, instead of a binary classification. We also calculated three psychologically meaningful parameters ( α , ν , and t0) instead of the RT. This study is the first to investigate the relationships among the above parameters.

We tested three hypotheses using computational models. The first hypothesis was informed by the previous finding that people tend to avoid negative situations rather than to show approach behavior to positive ones. Kahneman and Tversky (1979) explained this asymmetry between gain and loss using prospect theory. Katahira et al. (2011) revealed that the negative reward value of negative pictures was larger than the positive reward value of positive pictures. Thus, we expected the learning rate in the associative learning task to be higher in both younger and older participants for punishment trials than reward trials. In terms of developmental changes, Saito et al. (2020) reported that older participants showed markedly reduced sensitivity to positive expressions compared with younger participants. Thus, our hypotheses are as follows: 1–1. Learning rate parameters will be higher for punishment trials than reward trials. 1–2. Older participants will have low learning rates for rewards. In the visual search paradigm, we expected the younger group to exhibit superior performance compared with the older group (Salthouse, 2000). Accordingly, we hypothesized that each diffusion model parameter value will be higher in the younger participants than in the older ones. Finally, based on a straightforward interpretation of Saito et al. (2022b), we predicted that the ν in RT will be linked to the learning rates. The relationships between other parameters were investigated in an exploratory manner.

## 2 Methods

### 2.1 Participants

We recruited 29 young adult participants (13 women; mean ± SD age = 22.6 ± 2.1 years), all of whom were either undergraduate or graduate students at Kyoto University. We also recruited 32 older participants (16 women; mean ± SD age = 73.5 ± 5.3 years) from a local human resource center in Kyoto. All participants were paid for their participation. The sample size was determined by *a priori* power analysis, using a frequentist approach with an assumed α level because the data had been used in previous studies (Saito et al., 2022a,b). All participants provided written informed consent to take part in the study, which was approved by the ethics committee of the Unit for Advanced Studies of the Human Mind at Kyoto University and conducted in accordance with institutional ethical guidelines and the Declaration of Helsinki.

### 2.2 Stimuli

Seven grayscale images of male faces with neutral expressions were selected from a database containing 65 faces of Japanese individuals (Sato et al., 2019). One face was used as a distractor in the visual search task, and the others were used as targets in the associative learning and visual search tasks. The images were adjusted for brightness and contrast using Photoshop 5.0 (Adobe, San Jose, CA) and the mean luminance was equalized using MATLAB R2017b (MathWorks, Natick, MA). The stimuli were controlled in terms of both attractiveness and distinctiveness. The detection speed was not significantly different between the target faces in the visual search task according to the results of preliminary experiments (*F*(5,35) = 2.18, *p* = 0.079). All stimuli were cut into ellipses to exclude distinctive factors (e.g., hairstyle and facial contours), and had subtended visual angles of 3.5° horizontally × 4.5° vertically.

#### 2.2.1 Associative learning task

Three pairs of faces were used. The face pairs were fixed throughout the learning task. Each pair was allocated to one of the three value conditions (reward, punishment, or zero outcome), and this allocation was counterbalanced across participants. In the reward and punishment conditions, one face in each pair was designated as the target. Selecting the target resulted in a monetary reward (20 yen increase in each trial) or punishment (20 yen decrease in each trial) in 80% of trials (zero outcomes for the other 20%). The nontarget image in each pair was assigned the reverse probability pattern (i.e., 20% probability of monetary reward or punishment). In the zero-outcome condition, one face was the target, but the monetary outcome was always zero regardless of whether the participants selected the target or nontarget. This condition was set as a control condition to compare the learning conditions (reward and punishment). The target face statuses in each condition were counterbalanced across the participants. Each participant experienced 1 of 24 stimulus presentation combinations in the learning task.

#### 2.2.2 Visual search task

The same three face pairs from the associative learning task were used in the visual search task. The face classified as the target in each condition in the associative learning task was also the target in this task. An additional face with a neutral expression was used as a distractor. The search display was a square (11.0° × 11.0°) within which faces were presented in four positions at 4° intervals. One target face and three identical distractor faces were presented in each of the four positions in the target-present condition. Each target face was presented 8 times in each of the four positions, so that each target was presented 32 times in total. In the target-absent condition, the four identical distractor faces appeared in all of the positions.

### 2.3 Procedure

The participants were seated in a chair with a chin rest fixed 80 cm from the monitor. The experimental room was soundproofed and dimly lit.

#### 2.3.1 Associative learning task

The participants took part in a betting game, in which they were asked to choose a face from each stimulus pair based on their “gut feeling” and register their response by pressing the corresponding button on a response box. The goal of the task was to maximize their earnings. Because the participants were not told which of the paired faces was the target, they had to make a guess about which face would be more likely to lead to a reward. They were informed that the money they earned would be paid after the experiment, and were asked to try and earn as much money as possible. Each trial began with the presentation of a fixation cross for 500 m, followed by a pair of faces. The faces in the pair were positioned 2.5° above or below the fixation cross (0.9° × 0.9°), such that they appeared in the center of the screen. The positions of the target and nontarget faces were pseudo-randomized. After the participant made a response, a “reward” message (plus 20 yen, minus 20 yen, or 0 yen) was presented on the screen, and a sound indicated whether the answer was correct or incorrect (no sound for 0 yen). Then, the amount of money earned was displayed on the screen for 1,800 ms. Each face pair was presented 10 times (total of 30 trials) in one block, and there were 10 blocks in the main experiment, resulting in 300 trials. To prevent the consecutive presentation of identical face pairs in the same position, the presentation order of the face pairs was pseudo-randomized. Prior to the experiment, the participants completed 30 practice trials to familiarize themselves with the task.

#### 2.3.2 Visual search task

Before the experiment, the participants were informed that no monetary reward or punishment would occur in this task. In each trial, a fixation cross was shown for 500 ms, followed by a stimulus array containing four faces (Figure 1B) that did not have any facial movements (i.e., neutral faces). The participants were instructed to indicate whether one of the faces was dissimilar from the others, or if all four faces were the same, by pressing the corresponding button on the response box as quickly and accurately as possible. The allocation of the response buttons was counterbalanced across participants. Each block included 24 target-present trials (8 trials each in the reward, punishment, and zero-outcome conditions) and 24 target-absent trials. The main experiment consisted of four blocks, such that the total number of trials was 192. To prevent the consecutive presentation of identical targets in the same position, the order of trial presentation was pseudo-randomized in each block. There was no time limit. Before the experiment, the participants completed 24 practice trials.

In the associative learning task, two younger participants (−220 ~ −140 yen) and 10 older participants (−440 ~ −20 yen) had net negative amounts. Regardless of whether a negative or positive amount of money was earned, all participants received the same predetermined monetary bonus in the learning task (1,000 Japanese yen) after they had been debriefed.

### 2.4 Statistical approach

#### 2.4.1 Associative learning task

To determine whether the learning conditions affected face selection, and whether that effect differed depending on age, we performed three-way mixed ANOVA with factors of learning condition (reward, punishment and zero), trial block (1–20, 21–40, 41–60, 61–80, and 81–100), and age (younger and older). For *post-hoc* tests, *p*-values were adjusted using the Holm-Bonferroni sequentially rejective procedure (Holm, 1979). Learning was assumed to have occurred, if there was a performance difference between the first 20 and final 20 trials.

To build a reinforcement learning model, we established a modified multiple-armed bandit model (Hertwig et al., 2004; Ahn et al., 2017) based on the Rescorla-Wagner (delta) model in which the learning rates for reward and punishment were distinguished (Rescorla and Wagner, 1972). Because there was no feedback in the zero condition, the estimates were not uniquely determined; we used only the data for the reward and punishment conditions. Thus, we estimated four main parameters: the learning rate for reward ( $\eta_{r}$ ), learning rate for punishment ( $\eta_{p}$ ), inverse temperature for reward ( $\tau_{r}$ ), and inverse temperature for punishment ( $\tau_{p}$ ). First, we set four expected values for the four choices (target face choice in reward conditions, nontarget face choice in reward conditions, target face choice in punishment conditions, and nontarget face choice in punishment conditions). Next, we calculated prediction error (PE) by subtracting the monetary outcome (+1, 0, −1) from the expected value (EV) for each choice. After that, the following updating rule was formulated.


$$EV_{new}=EV_{old}+\eta_{r}*PE\;\mathrm{for reward}$$

$$EV_{new}=EV_{old}+\eta_{p}*PE\;\mathrm{for punishment}$$

To calculate the action probabilities, we used the softmax choice rule with the inverse temperature parameter ( $\tau_{r},\tau_{p}$ ), which reflects how individuals’ choices are made deterministically with respect to the value of the alternative choices (Kaelbling et al., 1996). An increase in the inverse temperature corresponds to a preference for model dependent choices, whereas a decrease in the inverse temperature reflects a tendency toward more random decisions. The learning rates represent the sensitivity of feedback in a learning task, where η close to 1 places more weight on recent outcomes.

In addition, we applied Bayesian hierarchical modeling (Lee and Wagenmakers, 2014), which can delineate individual differences and similarities among participants and thus enhance the accuracy of statistical inferences (Gelman and Hill, 2006; Driver and Voelkle, 2018; Namba et al., 2022). The number of iterations was set to 5,000, the number of burn-in samples to 5,000, and the number of chains to four. The R-hat value for all parameters was 1.0, indicating convergence of the four chains (Stan Development Team, 2020). The details of the model, including the prior distributions, are described in the Supplementary material.^1^

#### 2.4.2 Visual search task

To determine whether the learning conditions affected visual search performance (i.e., RT and accuracy), and whether that effect differed by age, we performed two-way mixed ANOVA including learning condition (reward, punishment and zero) and age (younger and older). Similar to the associative learning tasks, *p*-values were adjusted using the Holm-Bonferroni sequentially rejective procedure. We were concerned with the difference between the reward, punishment, and no feedback conditions in the target-present trials, rather than the difference between the target-present and absent conditions. Thus, we focused on the target-present condition in the visual search task.

To build the hierarchical drift-diffusion model, we applied a response-fitting model that use two outputs: same or dissimilar. We extracted the three main parameters from the behavioral data using the hierarchical drift diffusion model: α,ν , and t0. For parameter estimation, we calculated individual-level parameters (and the within-condition covariance structure) for α,ν in the different learning conditions (reward, punishment, and zero), and we calculated a population-level parameter for t0. z was fixed at 0.5. This was due to the need to constrain parameters for stable convergence (Kinosada, 2019). We determined whether the values of R-hat were close to 1 (values closer to 1 indicate greater convergence) and calculated the variation between the four chains (smaller variance reflects greater convergence). The number of iterations was set to 5,000, and the number of burn-in samples was set to 5,000. The diffusion model, including the prior distributions, was described in detail in (see Footnote 1).

Finally, we explored the correlations among the underlying parameters for each participant. Since no sample size calculation or *a priori* power analysis was conducted, we used conservative criteria, i.e., *r* > 0.30 (Cohen, 2013). All analyses were performed using R ver. 3.6.1 (R Core Team, 2019), with the “anovakun,” “bayesplot,” “brms,” “cmdstanr,” “posterior,” “rstan,” “tidyverse” packages (Bürkner, 2017; Gabry et al., 2019, 2023; Wickham et al., 2019; Stan Development Team, 2020; Bürkner et al., 2023; Iseki, 2023).

## 3 Results

### 3.1 Associative learning task

**Figure 2:**
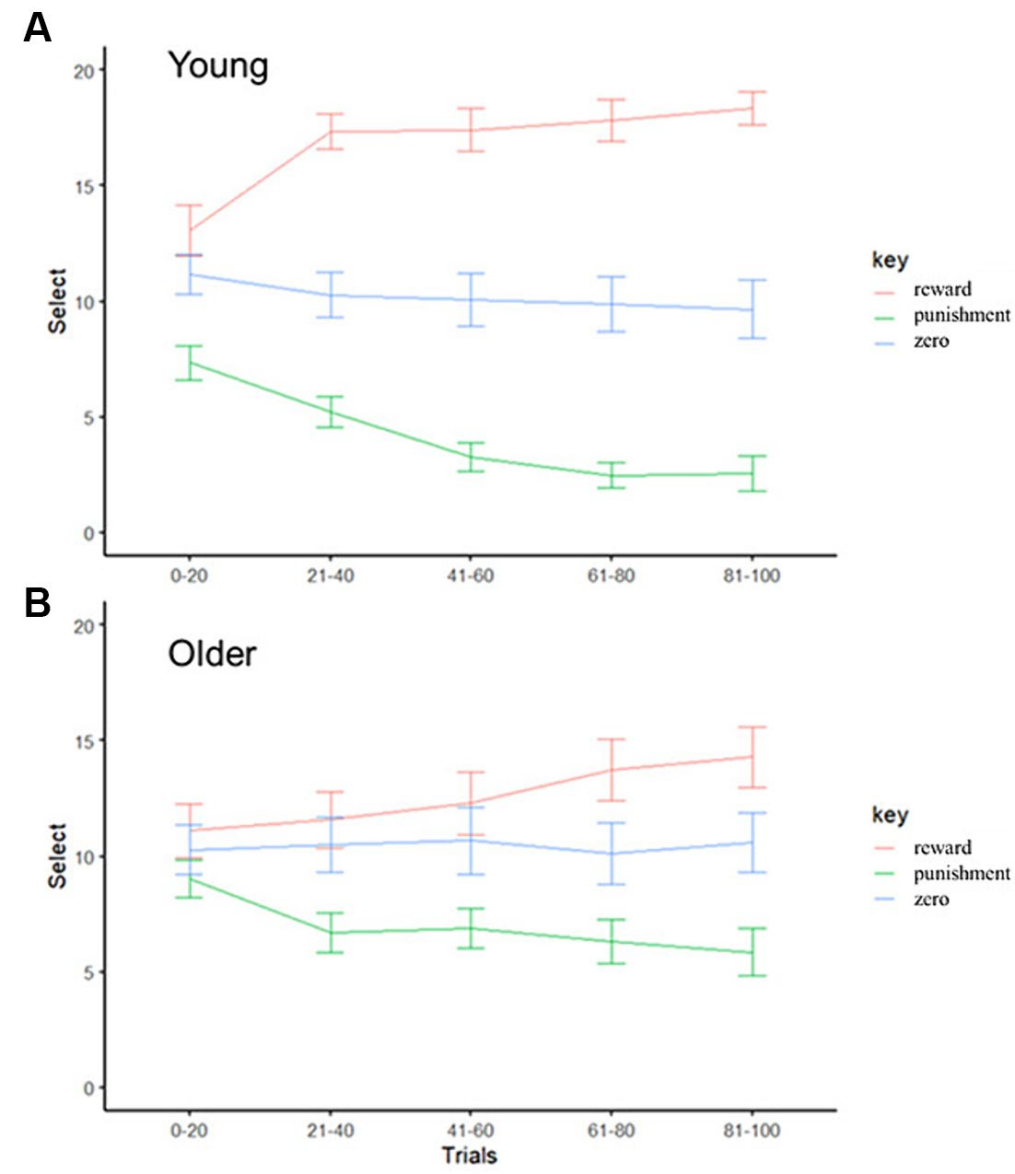
**(A)** Mean (± standard error) proportion of target faces selected by younger participants for each block (1–20) in the reward, punishment and zero conditions. **(B)** Mean (± standard error) proportion of target faces selected by older participants for each block (1–20 trials) in the reward, punishment, and zero conditions.

First, we checked the learning curves for the young and older participants, as shown in Figure 2. A visual inspection of the performance of the younger and older participants indicated good learning outcomes in both groups, although the younger participants exhibited more efficient learning. Three-way mixed ANOVA was performed to evaluate the effects of age, learning and trial block. There was no main effect of age or trial block, and interaction effect between age and trial block (*F*s < 0.35, *p*s > 0.55, *η_G_^2^*s < 0.002), but the main effect of learning condition was significant (*F*(2, 110) = 49.61, *p* < 0.001, *η_G_^2^* = 0.32). Participants in the reward condition performed significantly better than those in the zero and punishment conditions, and participants in the zero condition performed better than those in the punishment condition (*t*s > 4.36, *p*s < 0.001). There was also an interaction effect between trial block and learning condition (*F*(8, 440) = 13.43, *p* < 0.001, *η_G_^2^* = 0.05). The *post hoc* test showed significant differences between trial blocks in the reward and punishment conditions (*F*s > 14.22, *p*s < 0.001, *η_G_^2^*s > 0.06), but there was no difference between trial blocks in the zero condition (*F*(4, 220) = 0.38, *p* = 0.82, *η_G_^2^* = 0.00). In the reward condition, the selection rate was significantly higher in the last 20 trials than in the first 20 trials (*t* = 5.04, *p* < 0.001), but the opposite was true in the punishment condition (*t* = 5.30, *p* < 0.001). This indicates that associative learning for reward and punishment occurred in both participants. In addition, there was an interaction effect between age and learning condition (*F*(7, 43) = 7.43, *p* = 0.001, *η_G_^2^* = 0.07). The *post hoc* test showed significant differences between younger and older participants in the reward and punishment conditions (*F*s > 9.25, *p*s < 0.004, *η_G_^2^*s > 0.10), with the effects being smaller in older than younger participants, but there was no difference in the zero condition (*F*(1,55) = 0.02, *p* = 0.88, *η_G_^2^* = 0.00). Moreover, the performance of younger participants significantly differed among the conditions; performance was best in the reward condition, followed by the zero condition and finally by the punishment condition (*t*s > 5.40, *p*s < 0.001). Older participants performed worse in the punishment condition than the other two learning conditions (*t*s > 2.76, *p*s < 0.02), but the reward condition did not differ from the zero condition (*t* = 1.35, *p* = 0.19). In summary, the younger participants were able to learn reward and punishment contingencies in the associative learning task, and the older participants were able to learn punishment contingencies in the associative learning task but not reward contingencies.

**Figure 3:**
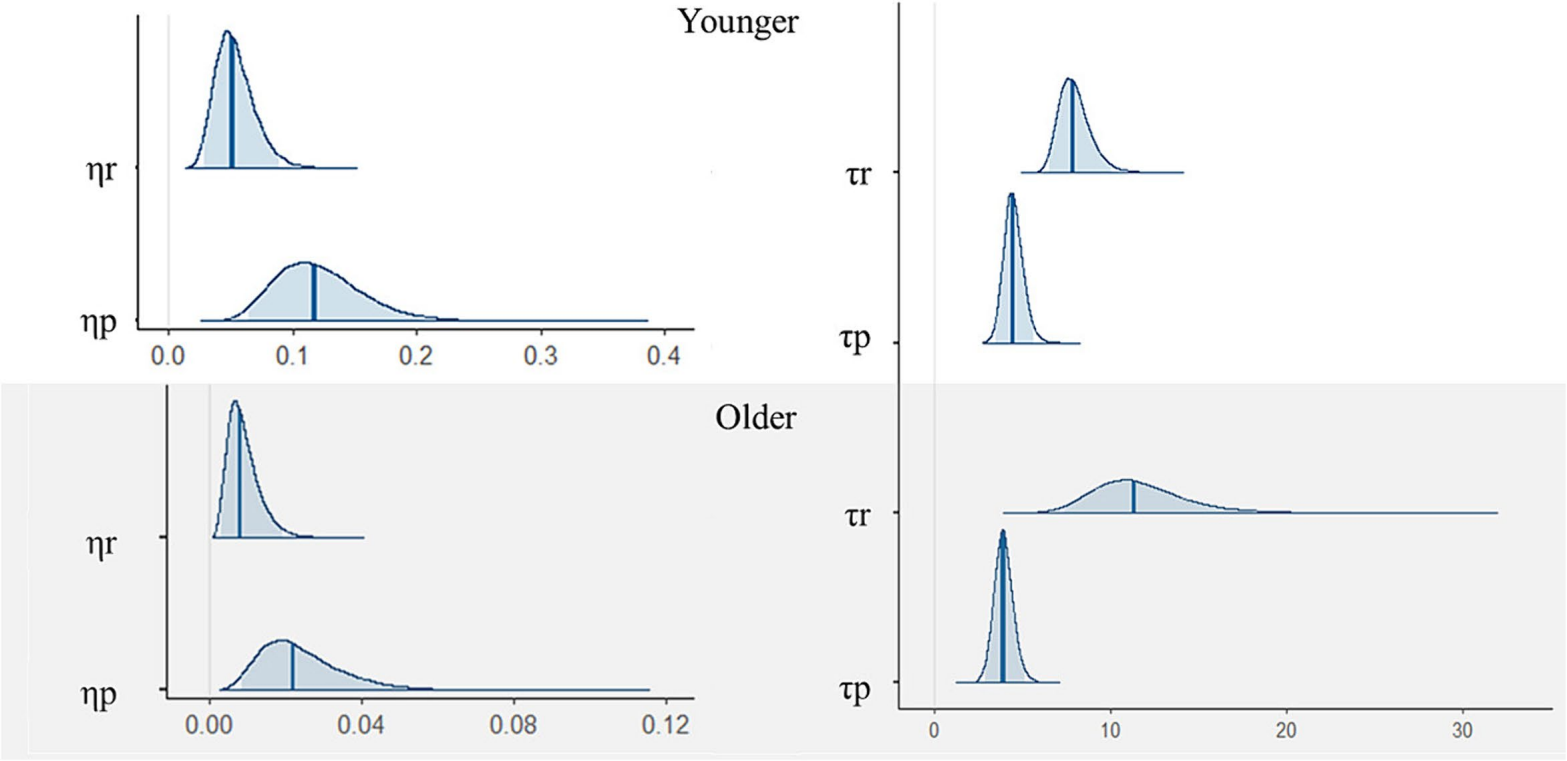
Posterior distributions of each parameter of the reinforcement learning model among younger (upper) and older participants (lower). Blue bars are expected *a posteriori* values and transparent blue regions are 95 credible intervals. η = the learning rates, τ = the inverse temperature, ∗*r* = for reward, ∗*p* = for punishment.

Next, as shown in Table 1 and Figure 3, we assessed the results for the reinforcement learning model parameters. There was a small difference in learning rate between the reward and punishment trials among the younger participants. Specifically, the learning rates were higher for punishment trials. For the inverse temperature parameters, both groups had higher values for the reward compared with punishment trials. We performed the posterior predictive check, comparing simulated and real data (Supplementary Figure S1^2^).

**Table 1:** All parameters in the reinforcement-learning model.

Name	Mean	95% Credible Intervals
Younger participants		
$\eta_{reward}$	0.05	[0.03, 0.08]
$\eta_{punishment}$	0.12	[0.07, 0.19]
$\tau_{reward}$	7.98	[6.71, 9.65]
$\tau_{punishment}$	4.50	[3.66, 5.46]
$\eta_{rew}-\eta_{pun}\;$	−0.07	[−0.14, −0.01]
$\tau_{rew}-\tau_{pun}\;$	3.48	[1.85, 5.35]
Older participants		
$\eta_{reward}$	0.01	[0.00, 0.02]
$\eta_{punishment}$	0.02	[0.01, 0.05]
$\tau_{reward}$	11.70	[7.98, 16.70]
$\tau_{punishment}$	3.95	[3.09, 4.89]
$\eta_{rew}-\eta_{pun}\;$	−0.02	[−0.04, 0.00]
$\tau_{rew}-\tau_{pun}\;$	7.75	[3.89, 12.80]

**Figure 4:**
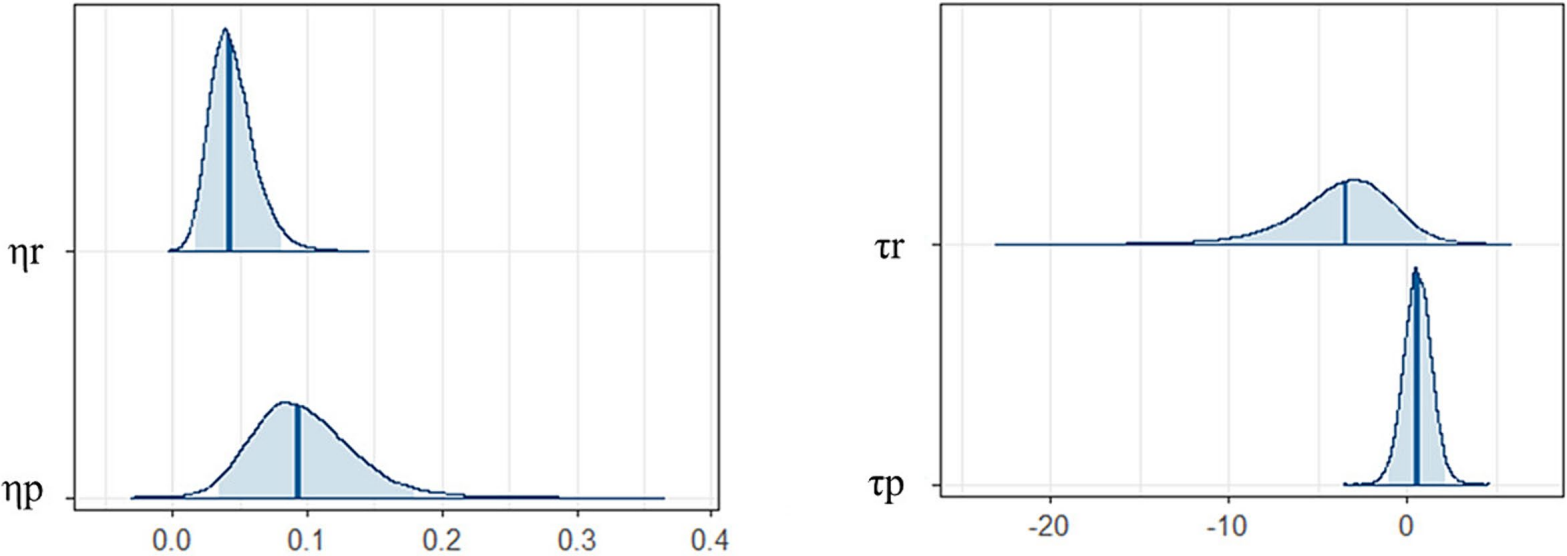
Posterior distributions of each parameter difference between younger and older participants. Blue bars are expected *a posteriori* values and transparent blue regions are 95% credible intervals. Positive values are a relatively large component of younger participants, while negative values are a relatively large component of older participants. η = the learning rates, τ = the inverse temperature, *r = for reward, *p = for punishment.

Figure 4 shows the posterior distributions of each parameter difference between younger and older participants. There were differences between the younger and older participants in learning rates but not inverse temperatures. Specifically, younger participants had higher learning rates than older ones (reward: mean of group difference = 0.04, 95% credible interval [CI] = [0.02, 0.07]; punishment: mean of group difference = 0.10, 95% CI = [0.04, 0.16]). There were no differences in inverse temperatures between the younger and older participants (reward: mean of group difference = −3.71, 95% CI = [−8.80, 0.38]; punishment: mean of group difference = 0.55, 95%CI = [−0.72, 1.84]).

### 3.2 Visual search task

To check the performance of the visual search task, we performed two-way mixed ANOVA including age and learning condition. It should be noted that this ANOVA analysis was a preliminary analysis to measure the tendency of the data. Regarding RT, there were main effects of age and learning condition (*F*s > 4.45, *p*s < 0.02, *η_G_^2^*s > 0.01). Younger participants (mean = 0.97) showed shorter RTs than older participants (mean = 1.58; *t* = 8.77, *p* < 0.001). Regarding the effect of learning condition, RT was significantly shorter in the reward condition than in the zero condition (*t* = 2.96, *p* = 0.01), and there was a trend toward a difference between the punishment and zero conditions (*t* = 1.78, *p* = 0.08). There was no interaction effect between age and learning condition (*F*(2, 110) = 2.03, *p* = 0.14, *η_G_^2^* = 0.005). Regarding accuracy, no significant main or interaction effects were found (*F*s < 0.80, ps > 0.45, *η_G_^2^*s < 0.006). In summary, in the visual search task, the younger participants were faster than the older ones. Moreover, the RT in the reward condition, but not in the punishment condition, was significantly different to that in the zero condition over the two age groups.

**Figure 5:**
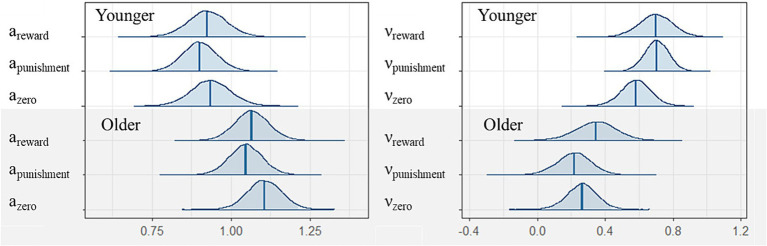
Posterior density plot of the group means of the six parameters as produced in the group (y = younger, o = older) and conditions (reward, punishment, and zero). Left: the threshold separation (α), right: the drift rate (ν).

As described above, we computed the three drift diffusion parameters (*α*, ν, t0) from the visual search data for the younger and older participants. Figure 5 shows the *α* values, i.e., the threshold of accumulated evidence for all conditions (two groups: younger and older; three conditions: reward, punishment, and zero). Visual inspection indicated no differences among the reward, punishment, and zero conditions, but there were differences between the younger and older participants only for the zero condition ( $a_{reward}$ : mean of group difference = −0.14, 95% credible interval [CI] = [−0.31, 0.02]; $a_{loss}$ : mean of group difference = −0.15, 95% credible interval [CI] = [−0.30, 0.01]; $a_{zero}$ : mean of group difference = −0.17, 95% credible interval [CI] = [−0.34, −0.01]). These results indicate that the older participants needed more information before they made a response regarding the value-driven unlearned faces. The posterior predictive check was further confirmed using reaction time values in the simulated and real data of younger and older participants [Supplementary Figure S2; (see Footnote 2)].

Figure 5 shows the results for ν, i.e., the speed at which the participants accumulated evidence before making a response. Even in the younger group, although visual inspection indicated that the positive and negative learning conditions had a relatively higher ν compared with the zero condition, performance did not differentiate positive and negative learning conditions from the zero condition ( $\nu_{rew\_to\_zero}$ : mean of group difference = 0.12, 95% credible interval [CI] = [−0.05, 0.28]; $\nu_{loss\_to\_zero}$ : mean of group difference = 0.12, 95% credible interval [CI] = [−0.07, 0.31]). There were differences between the younger and older participants ( $\nu_{reward}$ : mean of group difference = 0.35, 95% credible interval [CI] = [0.05, 0.66]; $\;\nu_{loss}$ : mean of group difference = 0.49, 95% credible interval [CI] = [0.24, 0.74]; $\nu_{zero}$ : mean of group difference = 0.32, 95% credible interval [CI] = [0.08, 0.57]).

t0 is based on all time components unrelated to the information accumulation process, as stated previously. There were differences between the younger and older participants ( $t0_{younger}$ : mean = 0.36, 95% credible interval [CI] = [0.35, 0.36]; $t0_{older}$ : mean = 0.50, 95% credible interval [CI] = [0.49, 0.52]; $t0_{diff}$ : mean of group difference = −0.15, 95% credible interval [CI] = [−0.16, −0.13]). In other words, older participants needed more time both before and after information accumulation than younger adults.

### 3.3 Relationships between associative learning and visual search parameters

To avoid the influence of outliers, we explored the Spearman’s rank correlation coefficients among the underlying parameters for each participant. We extracted the mode value (most probable value) for each individual parameter. Tables 2, 3 show the relationships between associative learning and visual search parameters. In younger participants, only the learning rate in rewarded associative learning trials was correlated with the ν for faces in the reward condition (*r* = 0.38, *p* = 0.04). However, there were no other correlations between the associative learning and visual search task parameters (|*r*|s < 0.35, *p*s < 0.07). For older participants, there were no correlations between the associative learning and visual search task parameters (|*r*|s < 0.32, *p*s < 0.10).

**Table 2:** Spearman’s rank correlation coefficients between the underlying mode parameters from the reinforcement-learning and drift diffusion models for younger participants.

Variable		$\eta_{r}$	$\eta_{p}$	$\tau_{r}$	$\tau_{p}$
a(r)	Spearman’s *r*	0.23	−0.05	0.20	−0.18
a(p)	Spearman’s *r*	0.13	−0.04	0.21	−0.14
a(zero)	Spearman’s *r*	0.16	−0.04	0.13	−0.19
v(r)	Spearman’s *r*	**0.38**	**0.32**	0.28	−0.07
v(p)	Spearman’s *r*	0.11	**0.34**	0.26	0.27
v(zero)	Spearman’s *r*	0.23	**0.35**	0.24	−0.05

The bold values mean significant effects or significant trends.

**Table 3:** Spearman’s rank correlation coefficients between the underlying mode parameters from the reinforcement-learning and drift diffusion models for older participants.

Variable		$\eta_{r}$	$\eta_{p}$	$\tau_{r}$	$\tau_{p}$
a(r)	Spearman’s *r*	−0.14	−0.14	−0.04	−0.29
a(p)	Spearman’s *r*	0.03	−0.06	0.09	−0.17
a(zero)	Spearman’s *r*	−0.01	−0.24	0.03	−0.11
v(r)	Spearman’s *r*	−0.11	0.19	−0.15	−0.14
v(p)	Spearman’s *r*	−0.01	0.28	−0.19	−0.12
v(zero)	Spearman’s *r*	−0.06	0.32	−0.28	−0.14

## 4 Discussion

This study explored the psychological processes underlying the rapid detection of faces with emotional meaning by investigating the relationship between associative learning data and visual search data. First, we found that the learning rates for reward and punishment were higher for younger than older participants (Figure 4). This was consistent with the simple learning performances revealed by Figure 2. The results also showed that learning rate parameter values were higher for punishment than reward trials in younger participants only. Older participants did not show different learning rates between reward and punishment trials. This result is consistent with previous studies showing that aging reduces sensitivity to negative faces and information (Mill et al., 2009; Zhao et al., 2016; Richoz et al., 2018). Our results suggest that the sensitivity of learning feedback might decrease with age.

Both the younger and older participants in this study showed higher inverse temperatures for reward than for punishment, and there was no difference between younger and older participants for this parameter (Figure 4). The inverse temperature parameter reflects the degree to which an individual retains their previous learning history (Katahira, 2015). Thus, both younger and older participants could have kept learning history and learning rate updates in the reward trials than punishment trials. However, the younger participants may have adjusted for this difference in sensitivity (i.e., higher learning rate for punishment than reward). Our reinforcement learning model shed light on the associative learning process in both younger and older participants.

For the visual search paradigm, ν was larger and t0 was smaller in the younger group than in the older group, consistent with our hypothesis. This result is consistent with Saito et al. (2022a), who revealed that RT in visual search tasks differs between younger and older adults. This finding further demonstrates that the younger group had superior efficiency in terms of information accumulation (ν) compared to the older group. Additionally, the perceptual time leading to motion onset (t0) was shorter in the younger group. Aging might reduce the speed with which information accumulates and attention is allocated to value-associated faces. Only in the zero condition, the older participants needed more information before they made a response (α) compared to the younger participants. This could be interpreted as that the older participant’s learning in the associative learning task affected the amount of information needed before they made a response, even if only slightly, so that they no longer differed from the younger participants. Figure 5 also shows that the threshold parameters for the value-driven conditions in the older participants were closer to the left than in the zero condition. The decline in performance among older participants is amenable to decomposition across distinct components as the current study indicated. The identification and elucidation of requisite interventions tailored to these specific components entail the pursuit of future research investigations.

We hypothesized that the ν for RT would be linked to each learning rate parameter. However, our results only partially supported this prediction, with the data from the younger participants in the reward condition. The result implies that sensitivity to reward in an associative learning task facilitates the accumulation of information in a visual search task for younger, but not older, participants. During an experiment in which a learning task and visual search were performed in relatively rapid succession, short term reward sensitivity was advantageous, although this effect diminished with age. This finding suggests that, in the context of building social relationships, more efficient accumulation of reward information is required in earlier stages of development. It is important to note that no such relationship was found between t0 and α . In the older participants, there were no correlations between the parameters from the two computational models. Thus, further research using a visual search task with more appropriate connections to associative learning tasks needs to be designed. Recently, Pedersen et al. (2017) attempted to combine the drift diffusion and reinforcement learning models. By directly applying the combined model to one learning task, we can expect to gain insight into the relationships between the mathematical parameters. The current study is the first to provide insight into how younger and older adults detect neutral faces that are associated with positive values.

### 4.1 Implications and future directions

Our findings have theoretical implications. The current results revealed the associations between computational parameters (i.e., learning rate and drift rate) underlying observable behavioral responses during value learning and value-driven detection of neutral faces in young participants. Several previous studies that used computational modeling have shown that the computational parameters reflect latent cognitive processes and are tightly linked to activity in specific brain regions. For example, a recent neuroimaging study reported that the drift rate estimated from face evaluation behaviors using a drift-diffusion model was associated with amygdala activity (Calabro et al., 2023). We expect our findings to provide insights into the neural mechanisms for value-driven detection of neutral faces and their aging patterns, which should be investigated in future computational neuroimaging studies.

Our findings may also have practical implications. A previous study has suggested that computational parameters classified clinical and non-clinical populations better than behavioral measures (Wiecki et al., 2015). A recent study reported that the RT performance of value-driven detection of neutral faces was associated with participants’ autistic traits (Saito et al., 2023). Collectively, our findings suggest that the computational modeling of the task may be helpful for the classification of individuals with autism spectrum disorder and those with typical development. Also, because the present study revealed the computational underpinnings of the effect of aging on value learning and value-driven detection of neutral faces, the same approach may provide information about pathological aging, such as dementia (cf. Irwin et al., 2018). Further research is warranted to test the task of value-driven detection of neutral faces and its computational modeling for clinical populations.

### 4.2 Caveats

This study had several limitations. First, the number of younger participants with successful learning outcomes was greater than that of older participants. The 8:2 feedback ratio used in the associative learning task was difficult for older adults, but may have been too easy for younger adults, thus making it difficult to compare associative learning performance in the visual search task. In the posterior predictive check [Supplementary Figure S1; (see Footnote 2)], the observed data for the older adults who failed to learn had a poor fit with the reinforcement learning model. This also poses a risk of the interpretability of the parameters of reinforcement learning models in the participants who failed to learn. Modification of the feedback ratio in the associative learning task or improving the reinforcement model, which can account for learning failures, is important for future research. The current study compared younger and older participants but factors other than age might contribute the observed differences/relationships. For example, attention is related to intelligence (Schweizer and Moosbrugger, 2004), and it is likely that young participants from Kyoto University and older participants from a local human resource center differ in attributes other than age, such as IQ (Chamorro-Premuzic and Furnham, 2008). The effects of age should be pursued with adequate control of factors that may cause interpretable outcomes. Future studies including older participants should use the Mini-Mental State Examination (Folstein et al., 1975) or other neuropsychological tests to explore whether general cognitive functions are preserved. In addition, the present study applied the diffusion model with a constrained number of trials (i.e., 32), which raises concerns about stable parameter estimates. Even when using only 24 trials, there was a sufficient correlation in the three-parameter diffusion model between the real and predicted values in the systematic simulation (Lerche et al., 2017). Notably, for detecting the condition differences, the noise of parameter estimation might not necessarily be critical. It also holds true that a larger trial count often improves parameter estimation, with the result that future research endeavors may demand the use of more trials and larger sample sizes to enhance the depth and scope of inquiry. Finally, as facial stimuli, the current study used only young male faces with neutral expressions, which may have biased the results considering that male faces tend to be viewed more negatively than female faces (Craig and Lee, 2020). Although emotional recognition performance for the facial expressions of older people is reportedly lower regardless of the observer’s age (Riediger et al., 2011), further studies are needed to determine whether this lower performance is applicable to older faces with neutral expressions.

## 5 Conclusion

In conclusion, we used reinforcement learning and drift diffusion models to compare the value learning process and value-driven detection of neutral faces between younger and older adults. The learning rates in the associative learning task, and the ν and t0 values in the visual search task, were higher in younger than in older participants. Sensitivity to learning feedback may decrease with age. During value-driven detection of neutral faces among young adults, we found that only the sensitivity to reward in the associative learning task promoted efficient accumulation of information during a visual search for neutral faces in younger but not in older adults. The parameter values of our mathematical model shed light on the contributing factors underlying the rapid detection of faces with emotional meaning in younger and older adults. Specifically, the sensitivity to feedback in the associative learning task, the speed of information accumulation and the perceptual time leading to motion onset in the visual search tasks, and the relationship between the speed of information accumulation and feedback sensitivity to reward, decreases with age. The current study underscored the significance of computational modeling in elucidating the cognitive process behind value-driven behavior and contributed to a deeper understanding of aging and related conditions, offering avenues for future investigation and potential interventions in both neuroscience and clinical contexts.

## Data availability statement

The datasets presented in this study can be found in online repositories. The names of the repository/repositories and accession number(s) can be found in the article/Supplementary material.

## Ethics statement

The studies involving humans were approved by all participants provided written informed consent to take part in the study, which was approved by the ethics committee of the Unit for Advanced Studies of the Human Mind at Kyoto University and conducted in accordance with institutional ethical guidelines and the Declaration of Helsinki. The studies were conducted in accordance with the local legislation and institutional requirements. The participants provided their written informed consent to participate in this study.

## Author contributions

SN: Investigation, Visualization, Writing – original draft, Writing – review & editing. AS: Conceptualization, Data curation, Formal analysis, Investigation, Methodology, Resources, Writing – review & editing. WS: Conceptualization, Funding acquisition, Project administration, Supervision, Validation, Writing – review & editing.

## Footnotes

^1^ https://osf.io/rw3e4/

^2^ https://osf.io/kdxz6
