# Supplementary material for: Computational analysis of value learning and value-driven detection of neutral faces by young and older adults
Source: Front Psychol. 2024 May 23;15:1281857. doi: 10.3389/fpsyg.2024.1281857 (PMC11153859; doi:10.3389/fpsyg.2024.1281857)
Supplement: Supplementary file 1 [file Data_Sheet_1.docx]

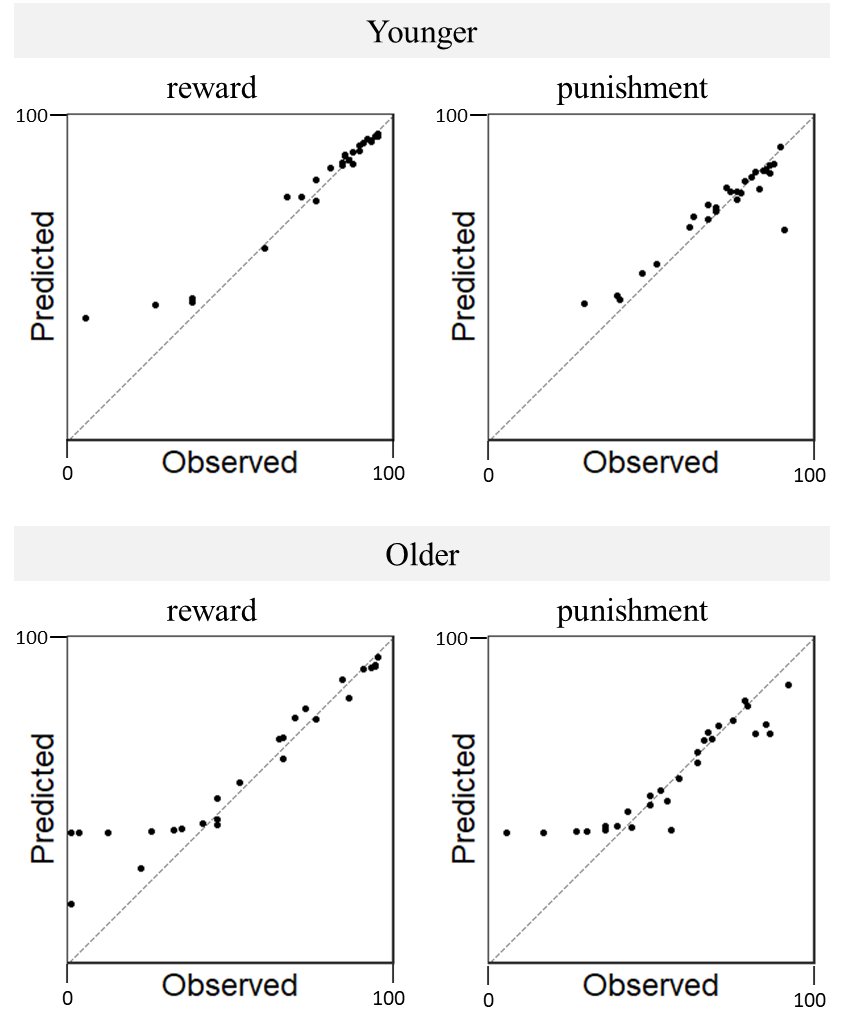


Figure 1S. Number of correct answers per individual for real data (x-axis) and simulated data using posterior parameters (y-axis) for younger and older participants.


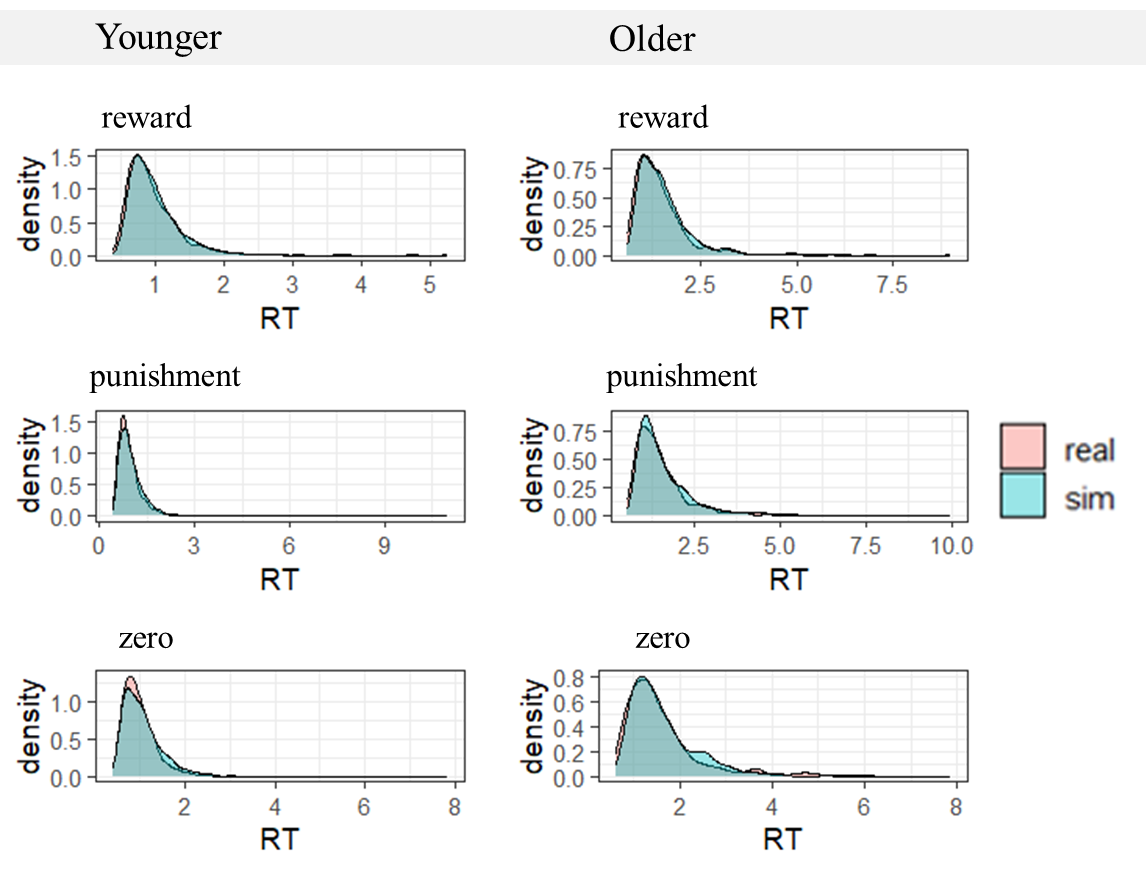


Figure 2S. Reaction times in younger and older participants. Distributions of reaction times in real data (red) and simulated data using posterior parameters (blue).
